# CD47 surface stability is sensitive to actin disruption prior to inclusion within the band 3 macrocomplex

**DOI:** 10.1038/s41598-017-02356-1

**Published:** 2017-05-22

**Authors:** Kathryn E. Mordue, Bethan R. Hawley, Timothy J. Satchwell, Ashley M. Toye

**Affiliations:** 1 School of Biochemistry, Biomedical Sciences Building, University Walk, Bristol, BS8 1TD United Kingdom; 2 Bristol Institute of Transfusion Sciences, NHSBT, Filton, BS34 7QH United Kingdom; 3 National Institute for Health Research (NIHR) Blood and Transplant Unit in Red Blood Cell Products at the University of Bristol, Bristol, BS8 1TD United Kingdom

## Abstract

CD47 is an important ‘marker of self’ protein with multiple isoforms produced though alternative splicing that exhibit tissue-specific expression. Mature erythrocytes express CD47 isoform 2 only, with membrane stability of this version dependent on inclusion within the band 3 macrocomplex, via protein 4.2. At present a paucity of information exists regarding the associations and trafficking of the CD47 isoforms during erythropoiesis. We show that CD47 isoform 2 is the predominant version maintained at the surface of expanding and terminally differentiating erythroblasts. CD47 isoforms 3 and 4 are expressed in all cell types tested except mature erythrocytes, but do not reach the plasma membrane in erythroblasts and are degraded by the orthochromatic stage of differentiation. To identify putative CD47 interactants, immunoprecipitation combined with Nano LC-MS/MS mass spectrometry was conducted on the erythroleukaemic K562 cell line, expanding and terminally differentiating primary erythroblasts and mature erythrocytes. Results indicate that prior to incorporation into the band 3 macrocomplex, CD47 associates with actin-binding proteins and we confirm that CD47 membrane stability is sensitive to actin disrupting drugs. Maintenance of CD47 at the cell surface was also influenced by dynamin, with sensitivity to dynamin disruption prolonged relative to that of actin during erythropoiesis.

## Introduction

**Figure 1:**
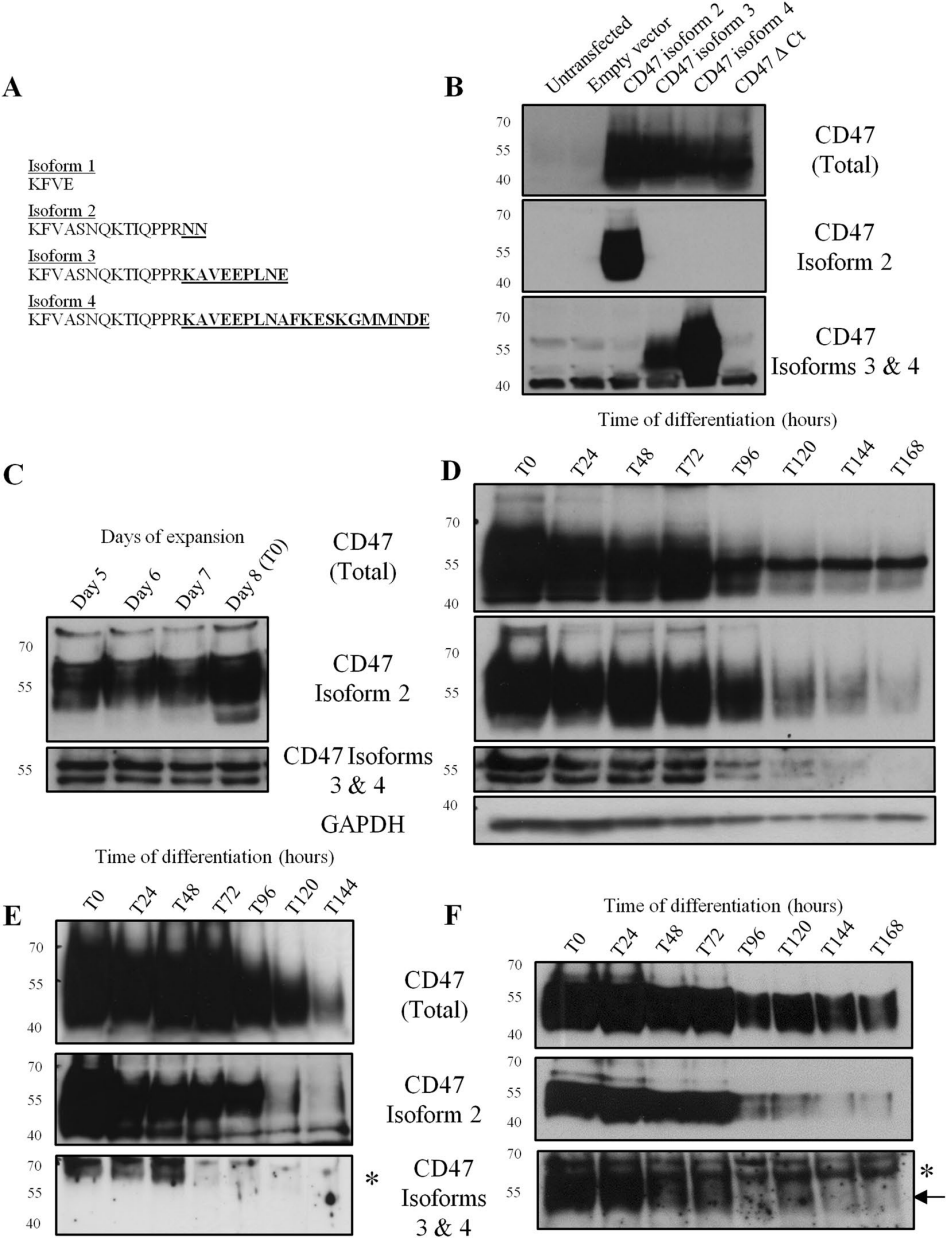
Only CD47 isoform 2 is detectable on the erythroblast surface during erythropoiesis. (**A**) The amino acid sequences of the 4 alternatively spliced isoforms of the CD47 C-terminus. (**B**) HEK293T cells were transfected with CD47 isoform 2, 3 and 4, and CD47 with a deleted C-terminus (Δ Ct) (5 µg DNA/10 cm^2^ dish). HEK293T cell lysates were separated by SDS-PAGE (10^6^ cells/lane) against the empty vector and untransfected cells. Western blots were probed with anti-CD47out1 (Total), anti-CD47 isoform 2, or anti-CD47 isoform 3 and 4 (n = 3). (**C**) Erythroblasts were harvested every 24 hours during expansion or (**D**) during terminal differentiation, lysed and then separated on SDS-PAGE (10^6^ erythroblasts/lane). Western blots were probed with anti-CD47out1 (Total), anti-CD47 isoform 2 or anti-CD47 isoforms 3 and 4. GAPDH was used as a loading control (n = 3). (**E**) Every 24 hours 5 × 10^6^ erythroblasts were incubated with BRIC126 to immunoprecipitate CD47 expressed on the surface, the eluate was then separated by SDS-PAGE. Western blots were probed with anti-CD47out1 (Total), anti-CD47 isoform 2 or CD47 isoforms 3 and 4 (n = 3). (**F**) 10 × 10^6^ erythroblasts were taken every 24 hours, lysed and then immunoprecipitated with BRIC126. The eluate was then separated on SDS-PAGE. Western blots were probed with anti-CD47out1 (Total), anti-CD47 isoform 2 or anti-CD47 isoforms 3 and 4 (protein band indicated by an arrow). *Indicates background band. (n = 2).

CD47 is a ubiquitously expressed ‘marker of self’ protein, originally identified as integrin-associated protein (IAP) following co-purification with β_3_ integrins from placenta^1, 2^. This ~52 kDa glycoprotein possesses an immunoglobulin variable (IgV) like N-terminal domain, five transmembrane domains and an alternatively spliced C-terminus that gives rise to four CD47 isoforms (Fig. 1A). CD47 isoform 2 mRNA is reported to be detected in haematopoietic and endothelial cells, whilst CD47 isoform 4 mRNA is expressed in neuronal cells^2–4^. CD47 isoform 3 is also found in neuronal cells and is thought to be involved in memory consolidation in the rat hippocampus^5^. Although CD47 isoforms exhibit tissue-specific distribution, the inhibitory receptor signal regulatory protein α (SIRPα), which suppresses macrophage activation and premature phagocytosis, can theoretically interact with all CD47 isoforms because they possess an identical IgV domain. The ‘marker of self’ function of CD47 was originally elucidated in murine erythrocytes^6^. Another established interaction involving the CD47 IgV domain is an interaction with the secreted glycoprotein thrombospondin-1 (TSP-1). CD47 ligation by TSP-1 induces activation of a subset of α_V_β_3_ integrin functions, including cell adhesion and chemotaxis, and α_IIb_β_3_ integrin, which is involved in platelet activation^7, 8^. The interaction of CD47 with integrins is dependent on the IgV and transmembrane domains of CD47^9–11^.

In non-erythroid cells, an association between the actin cytoskeleton and CD47 has been reported in T cells^9, 10, 12^, B cells^13^, and epithelial cells^14^, where it is involved in the spreading and motility of these cells, and also in the promotion of dendrite and axon growth in hippocampal neurons^15^. The IgV domain and transmembrane domain of CD47 are sufficient for the association with actin^16^. The direct interaction that mediates CD47 localisation within actin-associated complexes in these cells remains unknown although some downstream effectors following CD47 ligation have been elucidated, including PKC^10^, the Rho GTPases Cdc42 and Rac1^13, 15^, and Src kinases^14^; all of which regulate actin dynamics in favour of membrane ruffles and lamellipodia formation^17^. CD47 also reportedly associates with proteins linking IAP with cytoskeleton (PLICs^18^).

Mature erythrocytes express no integrins, instead CD47 is located within the Rh subcomplex (Rh, RhAG, GPB and LW), which is part of the larger band 3 macrocomplex (band 3, GPA, protein 4.2 and ankyrin-R). The efficient inclusion of CD47 into multi-protein membrane complexes is dependent on the presence of the complex linker, protein 4.2^19^. In the absence of protein 4.2, CD47 is reduced to ≤20% of normal levels^20–22^. We have previously found that during terminal differentiation CD47 is independent of protein 4.2 until the basophilic erythroblast stage (48 hours post-differentiation^20, 23^), suggesting that CD47 is dependent on alternative membrane stabilising interactions early during erythroblast development.

We set out to determine the associations required for CD47 membrane stability within the developing erythroblast prior to dependence on protein 4.2. We hypothesized that either 1) an alternative non-erythroid CD47 isoform is initially expressed that is not dependent on protein 4.2 expression but is instead lost by the basophilic stage of terminal differentiation, or 2) that CD47 associates with another component that provides surface stability before the expression and incorporation of CD47 isoform 2 into the band 3 macrocomplex. Here we use isoform specific antibodies to show that the main isoform expressed during erythropoiesis is CD47 isoform 2. We then use immunoprecipitation coupled with Nano LC-MS/MS mass spectrometry of early erythroblasts to show that prior to its incorporation into the band 3 macrocomplex, CD47 is indirectly associated with actin and its membrane expression is sensitive to actin disrupting drugs.

## Results

### Only CD47 isoform 2 is expressed at the cell surface during erythroid terminal differentiation

The specificity of the different CD47 isoform antibodies available to us (see Fig. 1A for C-terminal sequences) was confirmed using a panel of lysates prepared from untransfected HEK293T cells, HEK293T cells expressing the empty vector alone, or cells transfected with CD47 isoforms 2, 3, 4 or a version of CD47 with the whole C-terminus deleted (CD47 Δ Ct; Fig. 1B), which were separated by SDS-PAGE and then immunoblotted. Using a panel of human cell lines, primary cells and mature erythrocytes we confirmed that CD47 isoform 2 is restricted to haematopoietic and endothelial cells, being detected in Jurkat T cells, mature erythrocytes, K562 cells and HUVECs (Supplementary Fig. S1A). Interestingly, a band corresponding to CD47 isoforms 3 and 4 was detected in every cell line assessed except for mature erythrocytes (~55 kDa; Supplementary Fig. 1A) suggesting that expression of this isoform is not restricted to neuronal cells as originally proposed^4^.

**Figure 2:**
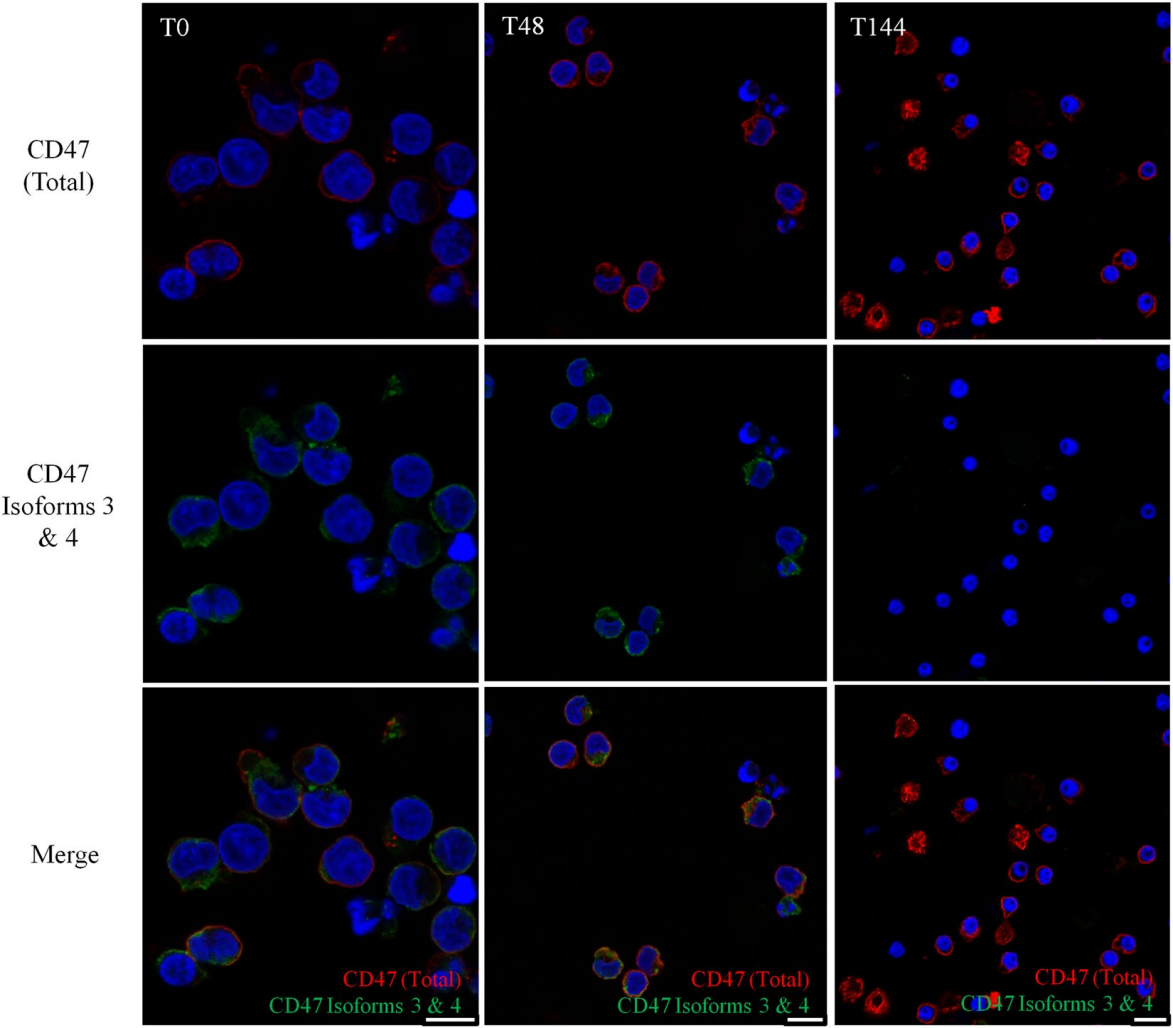
CD47 isoforms 3 and 4 are not detected at the surface erythroblast during terminal erythroid differentiation. Proerythroblasts (T0), basophilic erythroblasts (T48) and a mixture of late orthochromatic erythroblasts and reticulocytes (T144; 2 × 10^5^ cells/coverslip) were fixed in 1% PFA and 0.0075% glutaraldehyde, permeabilised in 0.05% Triton X-100, and then immunolabelled with BRIC126 (CD47 (Total)) followed by donkey anti-mouse Alexa™ Fluro 594, and CD47 isoforms 3 and 4 followed by donkey anti-rabbit Alexa™ Fluro 488. The nuclei were stained with DAPI (n = 2). Scale bar is 10 µm.

To determine CD47 isoform expression during erythroid terminal differentiation, an erythroid culture model was utilised to obtain lysates from expanding and terminally differentiating erythroblasts cultured from CD34^+^ cells^24^. CD47 isoforms 2, 3 and 4 are detected in expanding progenitors before terminal differentiation (Fig. 1C). Throughout terminal differentiation predominantly CD47 isoform 2 is expressed, whereas expression of CD47 isoforms 3 and 4 was detected until the late polychromatic erythroblast stage (96 hours post-differentiation; Fig. 1D). To determine whether CD47 isoforms 3 and 4 were expressed at the erythroblast membrane, every 24 hours CD47 at the erythroblasts surface was immunoprecipitated using BRIC126 and probed with isoform-specific antibody. CD47 isoform 2 was the predominant version detected at the erythroblast surface (Fig. 1E). CD47 isoforms 3 and 4 were not detected at the erythroblast surface (Fig. 1E), but these isoforms were detected until the late polychromatic erythroblast stage using total cell immunoprecipitation with BRIC126 (55 kDa, indicated by an arrow; Fig. 1F). CD47 isoform 2 was the predominant form detected by BRIC126 surface immunoprecipitation conducted on both K562 cells and SH-SY5Y cells (Supplementary Fig. S1B and C), and CD47 isoforms 3 and 4 were only detected in the surface immunoprecipitations of the neuronal SH-SY5Y cell line (Supplementary Fig. S1C). The absence of CD47 isoforms 3 and 4 at the surface was also demonstrated using confocal imaging (Fig. 2). In proerythroblasts (T0) and basophilic erythroblasts (T48) isoforms 3 and 4 were only detected in the cytoplasm, not at the surface, using confocal immunofluorescence. Late orthochromatic erythroblasts (T144) had no detectable immunofluorescence using the antibody specific to CD47 isoforms 3 and 4 (Fig. 2).

### Immunoprecipitation combined with proteomic analysis indicates a potential association between CD47 and the actin cytoskeleton early during erythroblast development

We utilized immunoprecipitation using an extracellular antibody to CD47 (BRIC126) coupled with Nano LC-MS/MS mass spectrometry to identify candidate binding proteins associated with CD47. Since CD47 expression becomes dependent on protein 4.2 during the basophilic stage of differentiation (48 hours post-differentiation^20^) CD47 was immunoprecipitated from expanding (EXP) erythroblasts (day 6 following CD34^+^ cell isolation), terminally differentiating proerythroblasts (T0) and basophilic (T48) erythroblasts. Immunoprecipitations were also conducted on K562 cells (which resemble erythroid precursors^25^) and mature erythrocytes. Data analysis involved selection of “clean” peptides, defined as peptides from the protein entries that were absent from the IgG isotype control immunoprecipitation (underlined and indicated bold; Table 1), or “enriched” peptides, i.e. peptides detected at greater than 2-fold compared to the IgG isotype control immunoprecipitation. Table 1 summarises the peptides of the proteins that were detected in example CD47 immunoprecipitations from 15 million K562 cells, EXP erythroblasts, T0 erythroblasts and mature erythrocytes. The T48 erythroblasts immunoprecipitation is not shown as no peptides were detected above the background IgG peptides. All peptides detected across all immunoprecipitations for the proteins listed that were below the two fold cut off are shown in Supplementary Table 1.

**Table 1:** Summary of the proteomic profile of peptides detected following BRIC126 immunoprecipitations in K562 cells, EXP and T0 erythroblasts, and mature erythrocytes (RBCs).

Accession Number	Protein Description	K562 cells			EXP			T0			Mature RBCs		
		Total	IgG	Ratio	Total	IgG	Ratio	Total	IgG	Ratio	Total	IgG	Ratio
P60709	Actin, cytoplasmic 1	71	33	2.2	242	109	2.2						
P61158	Actin-related protein 3	**5**	—	*				13	6	2.2			
O43707	Alpha-actinin-4	**8**	—	*	59	20	3.0	86	45	2.0			
Q562R1	β-actin like protein 2	14	3	4.7	45	19	2.4						
Q96C19	EF hand containing protein D2	**7**	—	*	12	3	4	15	6	2.5	**6**	—	*
P15311	Ezrin	12	6	2									
P47756	F-actin capping protein subunit β	7	1	7	17	6	2.8						
P21333	Filamin A	42	15	2.8	54	6	9						
Q08722	Leucocyte surface antigen, CD47	6	1	6	7	2	3.5	8	4	2	14	6	2.3
Q9UHB6	LIMA1				7	2	3.5	27	5	5.4			
P26038	Moesin	22	7	3.1	3	1	3						
O94832	Myosin-Id	12	2	6									
P35579	Myosin-9	228	72	3.2	374	166	2.3						
P35580	Myosin-10	154	37	4.2									
P12829	Myosin light chain 4	13	2	6.5	8	1	8	26	5	5.2			
P35241	Radixin	10	5	2									
P02549	Spectrin α chain, erythrocyte				**19**	—	*						
P11277	Spectrin β chain, erythrocyte				9	1	9						
Q9NYL9	Tropomodulin-3				**8**	—	*	8	2	4	2	1	2
P06753	Tropomyosin α-3 chain	9	1	9	3	3	1	**5**	—	*			
Q71U36	Tubulin α-1A chain				**14**	—	*	**23**	—	*			
P62988	Ubiquitin	19	3	6.3									
P68036	Ubiquitin-conjugated enzyme E2 L3	14	5	2.8							**4**		*
P08670	Vimentin				**5**		*	**2**		*			

The total Bric126 IP peptides and IgG peptides are shown alongside the ratio Total/IgG. Clean total peptides are underlined and bold. *Indicates no ratio as there were no IgG immunoprecipitated peptides.

As expected CD47 peptides were detected in all immunoprecipitations, but at a low abundance, likely due to the high degree of glycosylation of CD47 and the fact that membrane proteins are difficult to detect using mass spectrometry^26^. Actin, non-muscle myosin II proteins, actin-binding proteins (ABPs) and proteins involved in protein degradation via ubiquitination were immunoprecipitated with CD47 (Table 1). The association between CD47 and ABPs was observed particularly at the earlier timepoints during erythropoiesis, including EXP and T0 erythroblasts, and was also observed in K562 cells. The association of ABPs with CD47 was reduced by the basophilic erythroblast stage (T48; Supplementary Table 1). No peptides from band 3 macrocomplex proteins were detected, even though CD47 is known to be incorporated into this complex and is dependent on protein 4.2 from the basophilic stage^20–22^; this may reflect a weaker interaction between the two proteins in erythroblasts, or a dependence upon band 3 macrocomplex proteins via non-direct protein interactions. In addition, no integrin isoforms were detected in any of the CD47 immunoprecipitates. The plethora of ABPs in the CD47 immunoprecipitations suggests that CD47 may associate with the actin cytoskeleton, or is involved in regulating F-actin dynamics in erythroblasts similar to in other cells^10, 27^, but this is largely confined to the early erythroid development stage (Table 1).

### Plasma membrane stability of CD47 is reduced following disruption of F-actin remodelling

Although the CD47 immunoprecipitation using BRIC126 pulled down multiple ABPs, which is consistent with reports of CD47 indirectly interacting with actin in other cell types^13, 15^, it is difficult to pinpoint the specific interaction(s) responsible for an association with the actin cytoskeleton. Several putative interactions were explored including filamin A and filamin B via shRNA-mediated depletion, and vimentin via siRNA mediated depletion (data not shown) but the depletion of these proteins did not have a detectable effect on CD47. This could be because the proteins that we chose to investigate are not important to CD47 stability or that the cells compensate for the lost interactions in some unknown way.

**Figure 3:**
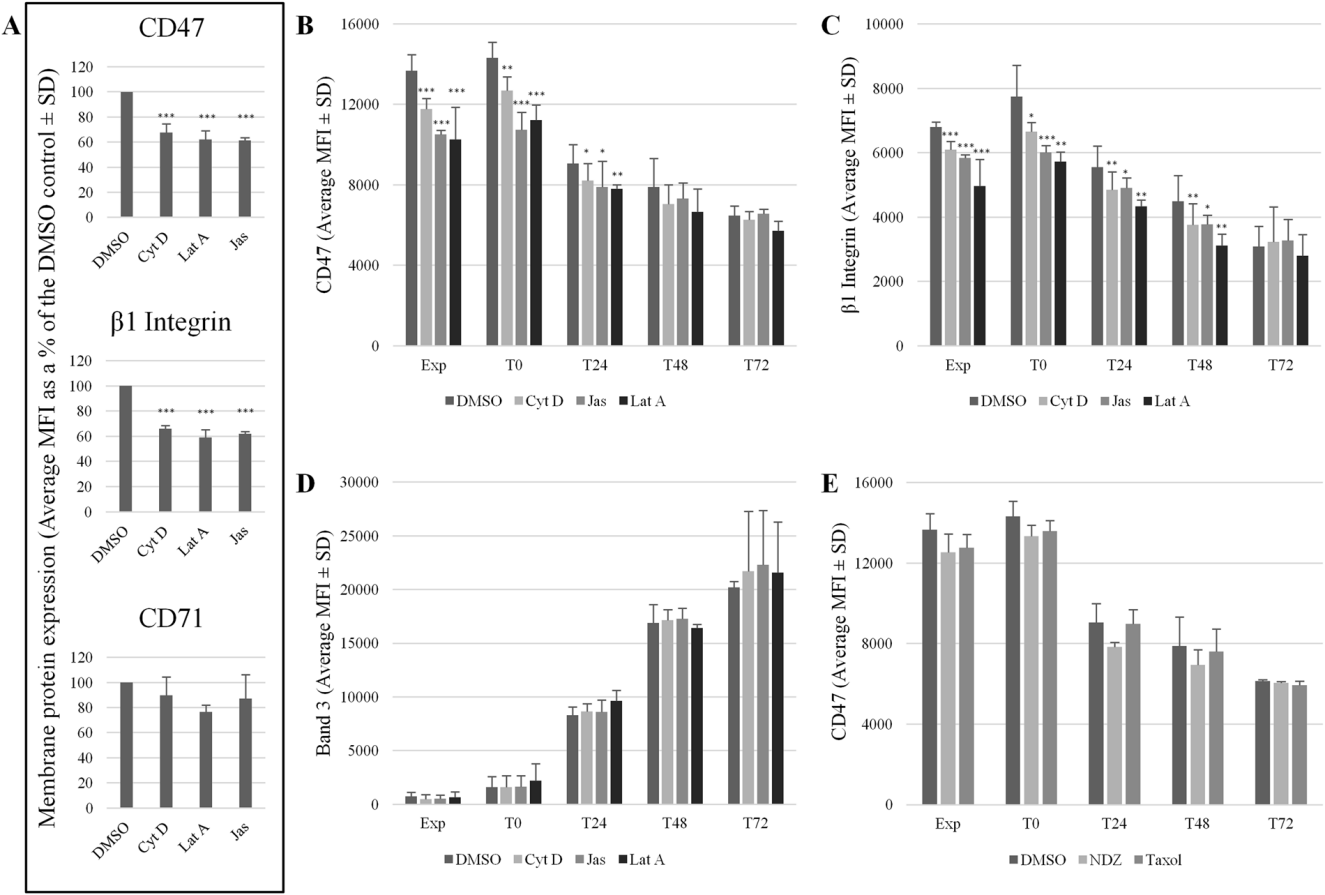
CD47 membrane stability is reduced following incubation with actin disrupting drugs. (**A**) K562 cells or (**B**–**D**) expanding (Exp) and differentiating (T0-T72) erythroblasts were incubated for 30 minutes with Cytochalasin (Cyt) D (5 µM), Latrunculin (Lat) A (1 µM), Jasplakinolide (Jas; 1 µM) or a DMSO control. (**E**) Erythroblasts were incubated with the microtubule stabilising drug paclitaxel (Taxol; 10 µM), the microtubule destabilising drug nocodazole (NDZ; 10 µM), or a DMSO control for 3 hours (n = 2). Treated cells were then incubated with BRIC32 (anti-CD47) and P5D2 (anti-β_1_ integrin). (**A**) CD71 (anti-Transferrin receptor) was used as a negative control in K562 cells (average MFI as % of the DMSO control ± SD (n = 3); ***p ≤ 0.001, **p ≤ 0.01, *p ≤ 0.05 using the Students *T* test). BRIC71 (anti-band 3) was used as a negative control in erythroblasts (average MFI ± SD (n = 3)). Cells were then incubated with an APC-conjugated monoclonal IgG1 anti-mouse secondary antibody. Emissions from 10,000 events were detected using a MACSQuant Analyser 10 flow cytometer and data was analysed using FlowJo version 10. The MFI were shown in (**B-D**) due to the differences in surface expression known to occur during terminal differentiation.

In the absence of a specific candidate binding protein, the response of CD47 stability in the plasma membrane to actin disrupting drugs was explored. This experiment was initially conducted on K562 cells (Fig. 3A). We hypothesised that should actin be involved in either the surface stability or trafficking of internal CD47 pools then there would be a disturbance of surface protein levels following actin disruption. To confirm involvement of actin and ABPs with CD47, the actin destabilising drugs Cytochalasin D (Cyt D) and Latrunculin A (Lat A), and the actin stabilising drug Jasplakinolide (Jas) were used^11^. Preincubation with actin disrupting drugs separately for 30 minutes caused a significant reduction in CD47 cell surface abundance compared to a DMSO control, as judged by flow cytometry, using the extracellular CD47 antibody BRIC32 (Cyt D 67.64% ± 7.02%; Lat A 62.04% ± 7.12%; Jas 61.15% ± 1.95%, *p* ≤ 0.001; (n = 4) Fig. 3A). Integrins are also known to be dependent on actin for their membrane trafficking^28^, so β_1_ integrin (P5D2) was used as a positive control and, similarly to CD47, was significantly reduced at the surface compared to the DMSO control (Cyt D 65.99% ± 2.72%; Lat A 58.92% ± 6.38%; Jas 61.81% ± 1.91%, *p* ≤ 0.001; (n = 4) Fig. 3A). The transferrin receptor (CD71) traffics independently of actin in K562 cells^11^ so was used here as a negative control. As expected, expression of CD71 on the K562 cell surface was unaffected by actin disruption (Fig. 3A).

The effect of actin disruption on CD47 surface levels was also assessed in *in vitro* cultured erythroblasts (Fig. 3B). Surface levels of β_1_ integrin were used as a positive control for actin disruption (Fig. 3C) and the anion exchanger band 3 was selected as the negative control (Fig. 3D) due to inconsistent and heterogeneous expression of CD71 within the primary cultured cells (data not shown). MFIs for each experiment are shown in Fig. 3 to illustrate alterations in surface abundance of the proteins that accompany terminal differentiation. Expressed as a percentage of control on each day, the level of CD47 at the surface of expanding erythroblasts was significantly affected by actin disruption compared to a DMSO control (EXP: Cyt D 86% ± 0.38%; Jas 76.84 ± 1.43; Lat A 74.84% ± 8.78 *p* ≤ 0.001 (n = 4)) and to a similar extent in proerythroblasts (T0: Cyt D 88.61% ± 1.41%; Jas 74.86 ± 2.05; Lat A 78.23 ± 1.35 *p* ≤ 0.01 (n = 4) Fig. 3B). From 48 hours of differentiation onwards the actin disrupting drugs had no significant effect on CD47 levels at the surface (T48: Cyt D 93.51% ± 8.42%; Jas 97.53 ± 11.98; Lat A 87.73 ± 3.26 *p* > 0.05 (n = 4) Fig. 3B).

Surface levels of β_1_ integrin mirrored those of CD47 during expansion (EXP: Cyt D 89.66% ± 3.33%; Jas 85.94 ± 1.02; Lat A 73.09 ± 9.7, *p* ≤ 0.001 (n = 4)) and at the beginning of terminal differentiation (T0: Cyt D 86.3% ± 7.02%; Jas 77.9 ± 5.63; Lat A 74.17 ± 3.71, *p* ≤ 0.01 (n = 4) Fig. 3C). However unlike CD47, β_1_ integrin remained sensitive to actin disruption until the polychromatic erythroblast stage (T72; Fig. 3C). Actin disruption had no effect on band 3 levels at any stage of erythroblast development (Fig. 3D). The results from the treatment with actin disrupting drugs on erythroblasts at different stages of erythropoiesis corroborate the results from the proteomic analysis (Table 1), in that actin and ABPs were found within CD47 immunoprecipitates in expanding erythroblasts and proerythroblasts, but were not found after this time point. Although peptides from α-tubulins and β-tubulins were detected in CD47 immunoprecipitations from EXP, T0 and T48 erythroblasts (Table 1 and Supplementary Table 1), microtubules stabilising and destabilising drugs paclitaxel (Taxol) and nocodazole (NDZ), respectively, had no effect of CD47 membrane stability following a 3 hour incubation at 37 °C (Fig. 3E).

**Figure 4:**
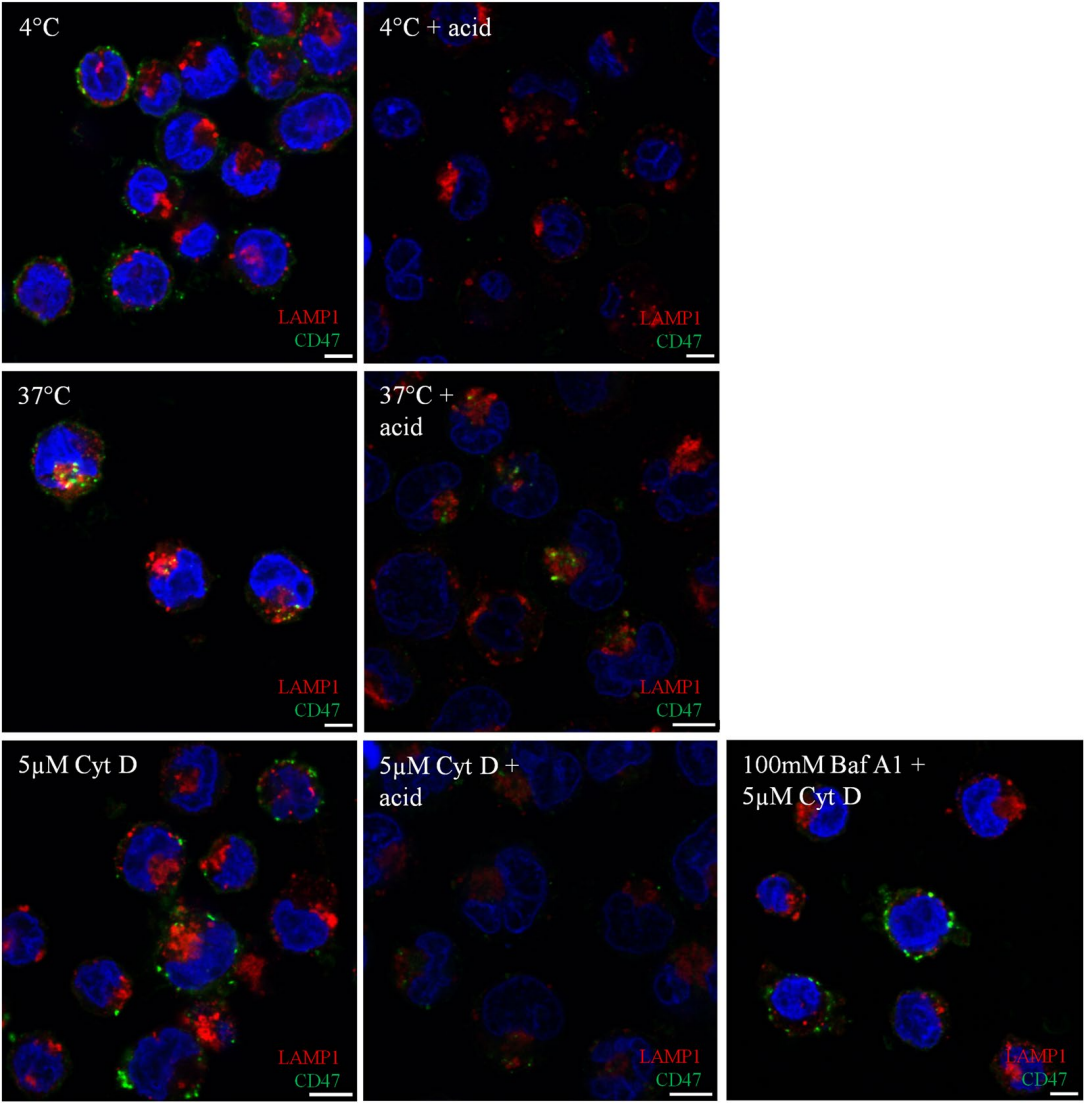
In K562 cells, CD47 cannot traffic through the endocytic pathway in the presence of Cytochalasin D and is trapped in membrane-proximal vesicles that are accessible to an acid wash. K562 cells (2 × 10^5^ cells/coverslip) were incubated with FITC-BRIC126 at 4 °C, before incubation at 37 °C ± Cyt D (5 µM). To study the involvement of lysosomal degradation, K562 cells were incubated with Bafilomycin (Baf) A1 (100 mM) for 1 hour at 37 °C prior to treatment with Cyt D. K562 cells were stripped (150 mM glycine [pH2]) to remove cell surface antibody, fixed in 1% PFA and permeabilised in 0.05% Triton X-100 before being probed with lysosomal-associated membrane protein-1 (LAMP-1) followed by donkey anti-rabbit Alexa™ Fluor 594. DAPI was used to stain the nucleus (n = 3).

Although the flow cytometric analysis would suggest that CD47 was lost from the surface following actin disruption in K562 cells and early erythroblasts (Fig. 3), we sought to identify where the internalised CD47 was localised following its loss from the cell surface. Using fluorescein isothiocyanate (FITC)–conjugated BRIC126 bound to CD47 on the K562 cell surface, we determined that following incubation at 37 °C for 30 minutes, FITC-BRIC126 was detected mainly within the perinuclear region within lysosome-associated membrane protein 1 (LAMP1) positive compartments (37 °C; Fig. 4). However, following a 30 minute preincubation at 37 °C with Cyt D (5 µM), there was no longer localisation to the perinuclear region and all FITC-BRIC126 was located in close proximity to the membrane (5 µM Cyt D; Fig. 4). Following an acid wash (150 mM glycine [pH2]), which strips accessible antibody bound at the cell surface^29^, very little FITC-BRIC126 was detected and any CD47 that had internalised was restricted to a compartment that was in close proximity to the plasma membrane (5 µM Cyt D+ acid; Fig. 4). The absence of detectable internalised FITC-BRIC126 was not due to rapid lysosomal degradation as there was no difference between cells treated solely with Cyt D, and cells treated with Cyt D but maintained in bafilomycin A1 (Baf A1 (100 mM); Fig. 4), which prevents lysosomal maturation and acidification^30^ and therefore blocks protein turnover in the lysosomes. The accumulation of FITC-BRIC126 at the surface and the accessibility of the acid wash suggested to us that full internalisation of CD47 was impeded by actin disruption, potentially resulting in the accumulation of CD47 within accessible endocytic structures as described previously^31, 32^.

### In the absence of protein 4.2 CD47 has a similar sensitivity to actin disruption

**Figure 5:**
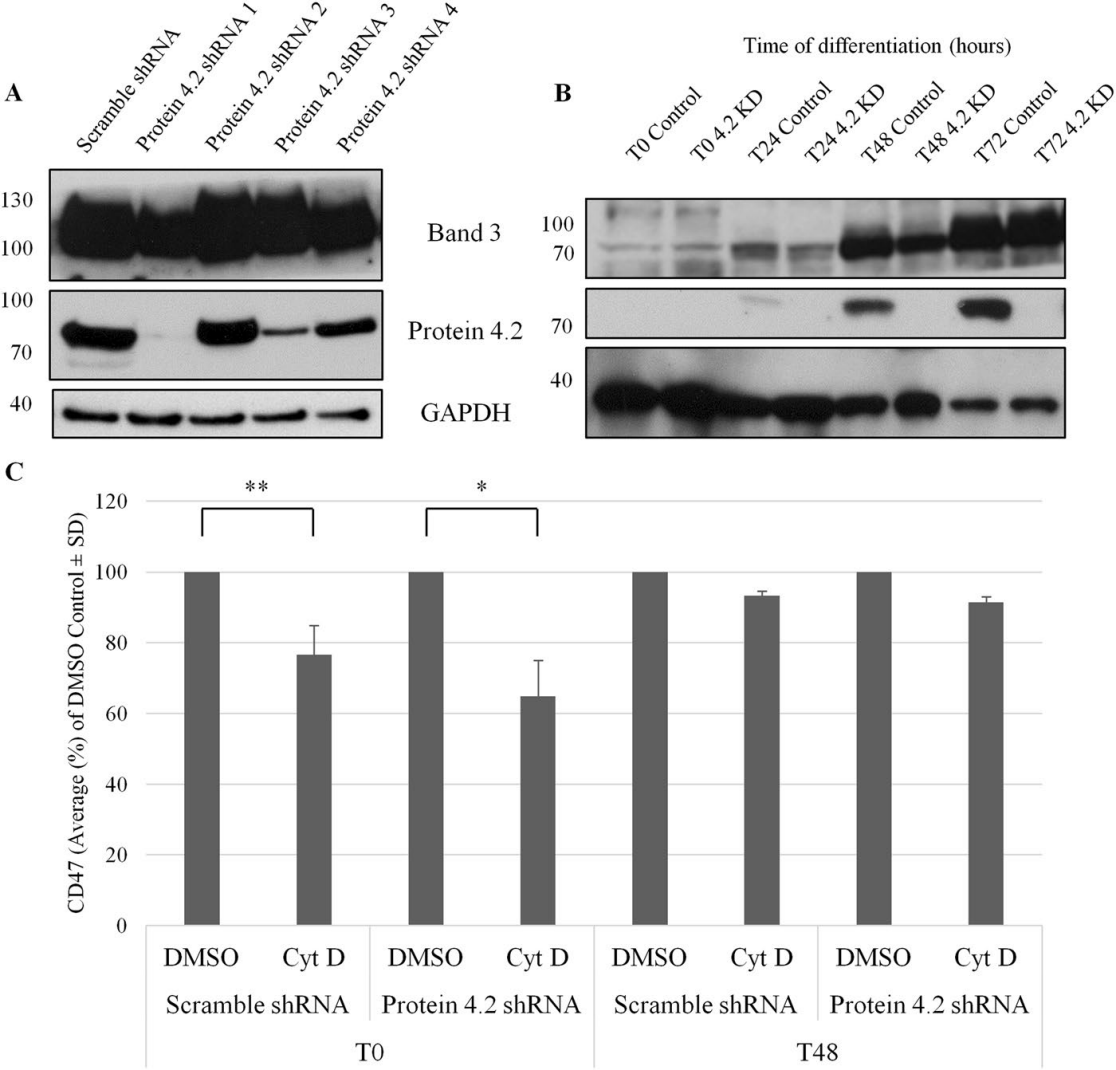
Sensitivity of cell surface CD47 to Cytochalasin D is unchanged in protein 4.2 deficient erythroblasts compared to control erythroblasts. (**A**) To assess the efficiency of the lentiviral pLKO.1 protein 4.2 shRNAs 1-4, transduced cells were differentiated until the polychromatic (T72) erythroblast stage, lysed and separated on SDS-PAGE (0.5 × 10^6^ cells/lane) against a Scramble control shRNA. (**B**) Erythroblasts, transduced with protein 4.2 shRNA 1 or the Scramble control shRNA, were taken every 24 hours during terminal differentiation, lysed and separated on SDS-PAGE (10^6^ erythroblasts/lane) (n = 4). Western blots were then probed with a rabbit anti-C terminal band 3 (YNTU), BRIC273 (anti-protein 4.2) and anti-GAPDH as a loading control. (**C**) Protein 4.2 deficient erythroblasts and scramble shRNA control erythroblasts, at T0 and T48, were incubated for 30 minutes with Cyt D (5 µM), or a DMSO control. Erythroblasts were then labelled with BRIC32 followed by APC-conjugated monoclonal IgG1 anti-mouse secondary antibody. Surface levels of CD47 were assessed by flow cytometry (Average MFI as a % of the DMSO control (n = 3); **p ≤ 0.01, *p ≤ 0.05 using the Students *T* test). 10,000 events were detected using a MACSQuant Analyser 10 flow cytometer and data was analysed using FlowJo version 10.

Protein 4.2 was next depleted using lentiviral transduction of protein 4.2 targeting-shRNA to confirm that the effects of actin disrupting drugs is independent of the presence of protein 4.2. First, the ability of four commercial protein 4.2 shRNA to deplete protein 4.2 was determined on erythroblasts (Fig. 5A); protein 4.2 shRNA1 was taken forward and was shown to have knocked down protein 4.2 expression throughout terminal maturation (Fig. 5B illustrates protein 4.2 expression up to 72 hours post-differentiation). Upon depletion of protein 4.2 the anticipated decrease in CD47 and increased levels of CD44 at the erythroblast membrane was observed (Supplementary Fig. S2A). These are the hallmark changes known to occur due to the absence of protein 4.2, confirming the importance of protein 4.2 for CD47 stability from the basophilic erythroblast stage onwards in a protein 4.2 patient sample^20^. Protein 4.2 remains depleted in filtered protein 4.2 shRNA treated reticulocytes compared to the scramble shRNA control reticulocytes (Supplementary Fig. S2B) and are accompanied by the alterations in CD47 and CD44 expression at the reticulocyte surface (Supplementary Fig. S2C), which is corroborated at the total protein level for CD47 and CD44 in the protein 4.2-depleted filtered reticulocytes compared to the scramble control filtered reticulocytes (Supplementary Fig. S2B).

CD47 levels in the Cyt D-treated protein 4.2-depleted erythroblasts at the beginning of erythroid differentiation were not significantly lower than that of CD47 in the scramble shRNA control erythroblasts when expressed as a percentage of control (T0: Scramble 76.66% ± 8.18% of DMSO control; protein 4.2 KD 64.83% ± 10.17% of DMSO control (*p* = 0.2); Fig. 5C). It should be noted that CD47 surface levels are increased by 34% ± 10.9% as a side effect of lentiviral transduction as reported previously^33^. However, as the reduction in CD47 at the proerythroblast surface, following treatment with Cyt D, is comparable to that observed in untransduced erythroblasts (T0 88.61% ± 1.41% compared to the DMSO control (Fig. 3B)), we can deduce that this is a non-protein 4.2 associated pool of CD47. Similarly, the membrane stability of CD47 in the protein 4.2 depleted basophilic erythroblasts treated with Cyt D was not significantly lower than the DMSO treated protein 4.2 depleted erythroblasts (T48: Scramble 93.34% ± 1.37% of DMSO control; protein 4.2 KD 91.43% ± 1.66% of DMSO control (*p* = 0.2)). It should also be noted that the population of CD47 remaining on the surface of the protein 4.2 deficient erythroblasts after 48 hours is severely reduced to 68.50% ± 6.27% (*p* = 0.001) of CD47 expressed at the surface of scramble shRNA control erythroblasts (Fig. 5C). This confirms that when protein 4.2 is substantially depleted, the interaction by the remainder of CD47 with the actin cytoskeleton is similar to normal erythroblasts during the initial stages of differentiation.

### Inhibition of endocytosis also reduces cell surface levels of CD47

**Figure 6:**
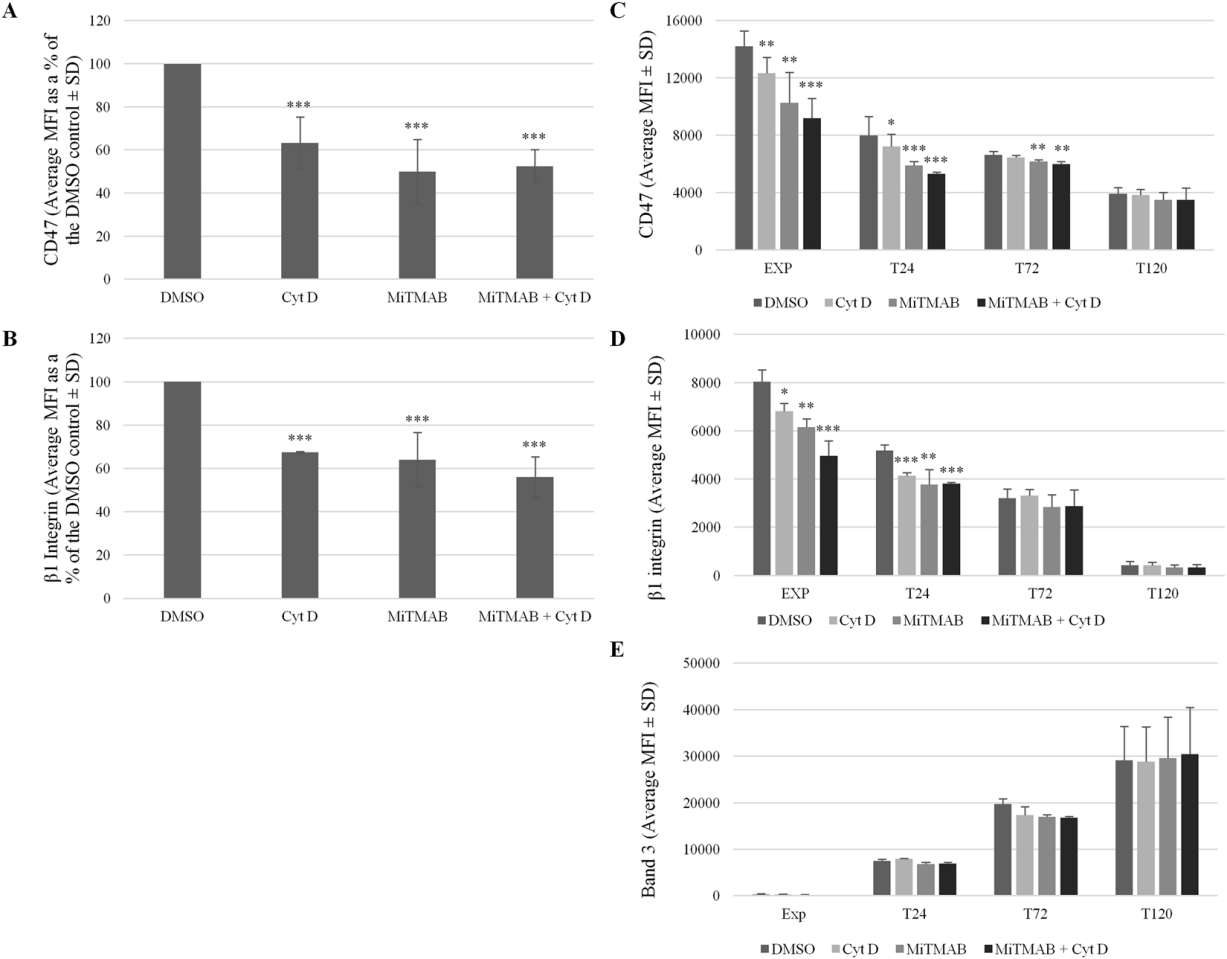
CD47 in K562 cells and erythroblasts is sensitive to MiTMAB and this occurs until the orthochromatic erythroblast stage (T120). K562 cells, expanding (EXP) and differentiating erythroblasts (T24, T72 and T120) were incubated with MiTMAB (30 µM) for 15 minutes prior to treatment with Cyt D (5 µM) for 30 minutes at 37 °C. K562 cells were then stained with (**A**) BRIC32 (anti-CD47) or (**B**) P5D2 (anti-β_1_ integrin) followed by APC-conjugated monoclonal IgG1 anti-mouse secondary antibody, before being assessed by flow cytometry (Average MFI as a % of the DMSO controls ± SD (n = 4)). Erythroblasts were stained with (**C**) BRIC32 (anti-CD47), (**D**) P5D2 (anti-β_1_ integrin) or (**E**) BRIC71 (anti-band 3) followed by APC-conjugated monoclonal IgG1 anti-mouse secondary antibody before being assessed by flow cytometry (Average MFI ± SEM (n = 2); ***p ≤ 0.001, **p ≤ 0.01, *p ≤ 0.05 using the Students *T* test). Emissions from 10,000 events were detected using a MACSQuant Analyser 10 flow cytometer and data was analysed using FlowJo version 10.

The known role for actin in endocytosis, and the accessibility of a proportion of CD47 proximal to the plasma membrane following actin disruption, led us to explore the effect that established endocytosis inhibitors have on CD47 membrane stability. Using a panel of inhibitors we identified two inhibitors that significantly reduced CD47 at the surface of K562 cells compared to a DMSO control (Supplmentary Fig. S3A). MiTMAB, an inhibitor that prevents dynamin GTPase from binding to the membrane^34^ by 49.96% ± 14.83% (*p* ≤ 0.001), and sucrose (72.13% ± 13.9% (*p* ≤ 0.05)) caused a pronounced reduction in surface availability of CD47. Both MiTMAB and sucrose inhibit clathrin-mediated endocytosis^34^, but Dynasore – another dynamin GTPase inhibitor, and Pitstop2 – a specific inhibitor of clathrin-mediated endocytosis^35^, had no significant effect on CD47 membrane stability in our hands (Supplementary Fig. S3A). K562 cells were co-incubated with Cyt D after pretreatment with MiTMAB (30 µM). There was no additive effect of actin disruption on the CD47 which remained at the surface (Fig. 6A). MiTMAB had a similar effect on β_1_ integrin surface levels (63.9% ± 12.64% (*p* = 0.05); Fig. 6B). This suggests that β_1_ integrins and CD47 are both sensitive to actin disruption (Fig. 3) and both require dynamin GTPase for their endocytosis (Fig. 6).

Expanding and differentiating erythroblasts were also incubated with MiTMAB in the presence and absence of Cyt D. Similarly to CD47 in K562 cells, CD47 was significantly reduced at the surface during terminal differentiation (T0: MiTMAB 72.32% ± 9.38%; MiTMAB + Cyt D 64.59% ± 4.90%; (n = 3; Fig. 6C). However, unlike treatment with actin disruptors where CD47 becomes insensitive at T48 (T48: Cyt D 91.90% ± 7.31% (*p* = 0.4); MiTMAB 76.51% ± 21.95% (*p* = 0.005); MiTMAB + Cyt D 70.73% ± 20.63% (*p* = 0.002) (n = 3)), a proportion of CD47 appears to remain sensitive to MiTMAB until the orthochromatic erythroblast stage (120 hours post-differentiation; Fig. 6C) suggesting that CD47 is still being actively endocytosed until this stage. Therefore, the sensitivity of CD47 to actin disruptors cannot simply be explained by a general disruption of endocytosis as there is a prolonged sensitivity of CD47 to endocytic disruption but not to actin disruptors. This suggests that although actin is involved in endocytosis^36^, actin and dynamin have temporarily distinct effects on the trafficking and membrane stability of CD47. In contrast, expression of β_1_ integrin at the erythroblast membrane became insensitive to the endocytic inhibitor at 72 hours post-differentiation (Fig. 6D); this is identical to the time point that β_1_ integrin becomes insensitive to treatment with Cyt D (Fig. 3C). Band 3 levels were unaffected by treatment with MiTMAB at all stages of terminal erythroid differentiation (Fig. 6E).

## Discussion

It is surprising that despite the known importance of CD47 as a ‘marker of self’^6^, which is known to be upregulated on stem cells and leukaemic cells^37^, little is known about CD47 isoform expression, intracellular interactions and trafficking in haematopoietic and erythroid cells. In this manuscript, we have shown that CD47 isoform 2 is the predominant isoform expressed on the surface of expanding and differentiating erythroblasts. We have also demonstrated that the maintenance of CD47 at the cell surface is dependent on actin and dynamin, but at temporally distinct stages.

Interestingly, CD47 isoform 3 and 4 were detected at the protein level in K562 cells and in *in vitro* cultured erythroblasts until the late polychromatic erythroblast stage (72–96 hours post-differentiation; Figs 1 and 2). It is at this stage during terminal differentiation that erythroblasts have been shown to lose the secretory pathway^38^, which could explain the observed loss of CD47 isoforms 3 and 4 after this stage. These isoforms were not detectable at the surface of erythroblasts (Fig. 1E) or K562 cells using immunoprecipitation, but were detected at the surface of the SH-SY5Y neuroblastoma cell line (Supplementary Fig. S1B and C). Therefore we propose that the expression of CD47 isoforms 3 and 4 during erythropoiesis is likely redundant.

In an attempt to find candidate proteins that interact with CD47 during erythropoiesis, trypsin treated peptides from BRIC126 co-immunoprecipitations were identified using sensitive Nano LC-MS/MS mass spectrometry. Peptide fingerprinting analysis of the CD47 immunoprecipitates provided an extensive list of putative associated ABPs in early erythroblasts (Table 1) suggesting that during expansion and the early stages of differentiation CD47 is associated directly, or indirectly via ABPs, with the actin cytoskeleton. This is consistent with previous observations by ref. 25 who reported that in K562 cells and erythroid precursors, in the absence of band 3, CD47 had cytoskeletal connectivity at 64% and 72%, respectively, following extraction by non-ionic detergents. CD47 in erythroblasts may also be involved in directly modulating actin dynamics in response to external stimulus through a mechanism similar to that demonstrated for CD47 in T cells and endothelial cells, where CD47 engagement with SIRP family members induced actin rearrangement via localisation to membrane lipid rafts^10, 27^. Further study is required to elucidate whether there is an actual candidate ABP that directly links CD47 to the actin cytoskeleton.

Following incubation with actin disrupting drugs, the surface level of CD47 was significantly reduced on K562 cells, where the surface abundance of CD47 reduced by up to 36%. CD47 was also reduced by 13% from the surface of erythroblasts during expansion and early during differentiation, following a 30 minute preincubation. This suggests that a dynamic actin cytoskeleton is required for normal trafficking and plasma membrane stability of CD47. It is notable that the loss of CD47 after treatment with actin disrupting drugs differs from observations reported for other membrane proteins where F-actin disruption caused an increase in abundance of membrane proteins at the cell surface^36, 39, 40^. We speculate that the difference in CD47 abundance at the cell surface of the erythroleukaemic K562 cell line compared to erythroblasts following actin disruption may be explained by the observation that cancer cells exploit CD47 to evade immune detection by macrophages^37^, therefore K562 cells may upregulate CD47 expression by altering expression levels or ABP associations.

Disruption of F-actin with Cyt D, Lat A and Jas significantly disturbs CD47 membrane stability in expanding and differentiating erythroblasts, and in K562 cells. We propose that actin disrupting drugs result in CD47 becoming disassociated from the cytoskeleton and then being subjected to incomplete endocytosis. Actin disruption with Latrunculin B, or expression of dynamin GTPase mutants, is known to reduce the number of scission events and increase the number of omega-shaped, and tubulated, clathrin-coated pits, with wider and less constricted necks^32, 41, 42^. The presence of open membrane invaginations would explain the accessibility of the acid wash following the incomplete internalisation of FITC-BRIC126 in Cyt D treated K562 cells (Fig. 4). Therefore, we suggest that our results with actin disrupting drugs likely underestimates the disruption of CD47, as the drugs enable CD47 internalization but also disturb the process of endocytosis.

In erythroblasts undergoing differentiation we have demonstrated that at the basophilic stage (48 hours post differentiation) CD47 becomes largely insensitive to actin disrupting drugs (Fig. 3B), which correlates with a reduction of ABPs in the proteomics of CD47 immunoprecipitates (Table 1 and Supplementary Table 1). This is the stage that CD47 is known to become dependent on protein 4.2 for its membrane stability^20^. Using shRNA mediated knock down of protein 4.2 via lentiviral transduction, we show that the residual pool of CD47 is sensitive to actin disruption to a similar level as in the presence of 4.2 (Fig. 5C). There was no increase in the proportion of CD47 that was lost from the surface in the presence of actin destabilizing drugs but this is likely because the population of CD47 that is dependent on protein 4.2 is already absent from the surface, leaving either an actin-associated pool or one that is being actively removed and redelivered to the surface.

In summary, we have demonstrated that CD47 isoform 2 is maintained throughout terminal erythroid differentiation excluding switching of CD47 isoform expression at the cell surface. During expansion and at the onset of terminal differentiation CD47 is associated, directly or indirectly, with the actin cytoskeleton until the basophilic stage (48 hours post-differentiation) when it is known to become associated with the band 3 macrocomplex^20^.

We also provide candidate interactors for CD47 in erythroblasts and K562 cells. Whilst we were unable to directly attribute CD47 membrane stability or intracellular trafficking to individual ABPs or proteins within this CD47 interactome, we anticipate that this interaction list will provide a useful resource for future studies.

Finally, in addition to providing an insight into the intracellular associations mediated by CD47 during erythropoiesis, the observed sensitivity of CD47 to actin cytoskeleton disruption in early haematopoietic progenitor cells and erythroblasts may be exploitable for the development of therapeutics that render leukemic cells more susceptible to phagocytosis.

## Materials and Method

### Antibodies

Monoclonal antibodies used were BRIC32, BRIC126 and fluorescein isothiocyanate (FITC)-BRIC126 (CD47), BRIC222 (CD44) BRIC273 (protein 4.2), BRIC71 (band 3; IBGRL, Bristol, UK), P5D2 (β1 integrin; Developmental Studies Hybridoma Bank, University of Iowa), PE conjugated transferrin receptor (CD71; BD Biosciences), CD71 (sc7327), anti-glyceraldehyde 3-phosphate dehydrogenase (GAPDH; Sigma-Aldrich), a C-terminal band 3 antibody (YNTU^24^) and a previously reported anti-lysosomal associated membrane protein 1 (LAMP1 270c^43^). The unpublished rabbit polyclonal anti-CD47 isoform 3 and 4 specific antibody and the previously described polyclonal antibody recognising the extracellular domain to CD47 (CD47out1^20^) were made by Dr. William Mawby, University of Bristol.

### Purification of the CD47 Isoform 2 Specific Antibody

The CD47 isoform 2 antibody was raised in a rabbit against the CD47 isoform 2 C-terminal peptide. The antibody was depleted of cross reactive antibodies against CD47 isoforms 3 and 4 using a peptide common to both isoforms (C-NQKTIQPPRKAVEEPLNE) coupled to a Sulfolink Resin Column (Thermo Scientific), according to the manufacturer’s instructions. Following this a purified CD47 isoform 2 specific antibody was then isolated using the C-NQKTIQPPRNN peptide coupled to a Sulfolink Resin Column.

### CD47 Isoform Constructs

CD47 isoform 2 (IMAGE clone: 2452556), isoform 3 (IMAGE clone: 2208344) and isoform 4 (IMAGE clone: 2437605) cDNA clones were ordered from The I.M.A.G.E. Consortium. CD47 isoform 2 cDNA was cloned into pMAX using BamHI and NheI. The C-termini of CD47 isoform 2 in pMAX (Lonza) was exchanged for CD47 isoforms 3 and 4 using XmnI and XbaI. The C-terminus of pMAX CD47 isoform 2 was removed, via the insertion of a stop codon at position 297 to generate CD47ΔCt, using QuikChange II Site-Directed Mutagenesis (Stratagene, Aglient Technologies), according to the manufacturer’s instructions. All constructs were fully sequenced (Eurofins Genomics, Germany).

### Mammalian Cell Culture

K562 erythroleukaemic cells and Jurkat T lymphocytes were maintained in IMDM (Life Technologies) supplemented with 10% foetal calf serum (FCS; GIBCO, Life Technologies) and penicillin/streptomycin (P/S; Sigma-Aldrich). SH-SY5Y neuroblastoma cells and human embryonic kidney (HEK) 293 T cells were maintained in DMEM (Life Technologies) supplemented with 10% FCS and P/S. Human Umbilical Vein Endothelial Cell (HUVEC) protein lysates were provided by Professor Harry Mellor (University of Bristol).

### Transient plasmid expression

HEK293T cells were transfected with pMAX containing CD47 isoform 2, 3 and 4, and CD47ΔCt, or with the empty pMAX vector by the calcium phosphate precipitation method and allowed to express for 48 hours before the transfected HEK293T cells were lysed in 1% NP-40 lysis buffer (20 mM Tris-HCl (pH8), 137 mM NaCl, 10 mM EDTA, 1% NP-40, 100 mM NaF, 10% glycerol, 10 mM sodium orthovanadate, 2 mM PMSF, 1% protease inhibitor cocktail set V (Calbiochem).

### Erythroid culture and erythrocyte samples

Waste peripheral blood mononuclear cells (PBMNCs) or mature erythrocytes were isolated from healthy donors following platelet apheresis (NHSBT, Filton), with informed consent for research use in accordance with the Declaration of Helsinki. Ethics approval for all experimental protocols was granted by Bristol Research Ethics committee (REC number 12/SW/0199) and all methods were carried out in accordance with approved guidelines. The culture system protocol used has been described previously^24^.

### Erythroblast Lentiviral Transduction

pLKO.1 protein 4.2 shRNA plasmids were designed by the Broad Institute and purchased from Open Biosystems (TRC number TRCN0000117232 gave the best protein 4.2 knockdown). Erythroblasts were transduced with lentivirus as described previously^44^. The reticulocytes generated from scramble shRNA transduced erythroblasts and protein 4.2 shRNA transduced erythroblasts were collected by filtration, using a 3 µm polycarbonate insert (Nunc, Thermo Scientific) as previously described^38^.

### Co-immunoprecipitation of CD47

For total cell immunoprecipitations K562 cells, SH-SY5Y cells, expanding and differentiating erythroblasts were lysed in 1% NP-40 lysis buffer, precleared for 1 hr using protein G beads (Thermo Scientific) and then incubated with BRIC126-labelled protein G beads for 1 hr, and then the beads were washed in lysis buffer. For surface immunoprecipitations, cells were incubated with BRIC126 (1 hr) before being lysed and incubated with protein G beads. For internal membrane protein immunoprecipitations, the cell lysate following the surface immunoprecipitation was incubated with BRIC126-bound protein G beads for 1 hr after the surface immunoprecipitation was conducted. Protein was eluted from the beads with SDS-PAGE sample buffer and then separated on SDS-PAGE.

### Proteomics

Proteomic analysis was conducted using Nano LC-MS/MS mass spectrometry by the University of Bristol Proteomics Facility. Total CD47 was immunoprecipitated using BRIC126 up to 15 million K562 cells, expanding (EXP) and differentiating erythroblasts (T0 and T48), and mature erythrocytes (n = 2). Briefly, protein G beads were fractionated by 1D SDS-PAGE until the dye front had moved approximately 2 cm into the separating gel. The gel lanes were cut into 2 equal portions and in-gel digested with trypsin. Extracted peptides were subjected to Nano LC-MS/MS mass spectrometry. The raw data files were processed using Proteome Discoverer software v1.4 (Thermo Scientific) and searched against the UniProt Human database (126385 entries) using the SEQUEST (Ver. 28 Rev. 13) algorithm. Peptide precursor mass tolerance was set at 10ppm, and MS/MS tolerance was set at 0.8 Da. Search criteria included carbamidomethylation of cysteine (+57.0214) as a fixed modification and oxidation of methionine (+15.9949) as a variable modification. Searches were performed with full tryptic digestion and a maximum of 1 missed cleavage was allowed. The reverse database search option was enabled and all peptide data was filtered to satisfy false discovery rate (FDR) of 5%. The Proteome Discoverer software generates a reverse “decoy” database from the same protein database used for the search and any peptides passing the initial filtering parameters that were derived from this decoy database are defined as false positive identifications. The minimum cross-correlation factor (Xcorr) filter was readjusted for each individual charge state separately to optimally meet the predetermined target FDR of 5% based on the number of random false positive matches from the reverse decoy database. Thus each data set has its own passing parameters.

### Immunoblotting

Cells were lysed in 1% NP-40 lysis buffer. Cell lysates were then separated by SDS-PAGE before being immunoblotted using the antibodies indicated in the figure legends, followed by HRP-conjugated rabbit anti-mouse or swine anti-rabbit secondary antibodies (Dako).

### Immunofluorescence

Differentiating erythroblasts were fixed in 1% PFA and 0.0075% glutaraldehyde in PBSAG, allowed to adhere to poly-L-lysine coated coverslips, permeabilised in 0.05% Triton X-100, blocked in PBS containing 4% BSA and then labelled with BRIC126 (total CD47) and CD47 isoform 3 and 4 antibody, followed by incubation with rabbit anti-mouse Alexa 594, and swine anti-rabbit Alexa 488 (Life Technologies), respectively. DAPI was used for nuclear staining. Single slice confocal images were taken using Leica SP5 confocal microscope (63X/1.4NA oil immersion lens). The protocol for labelling and uptake of cell-surface proteins using FITC-conjugated antibody and subsequent co-staining for LAMP1, have been reported previously^43^.

### Actin and Microtubule Disruption and Inhibition of Dynamin-Dependent Endocytosis

K562 cells and erythroblasts were treated with 5 µM Cytochalasin D, 1 µM Latrunculin A (Sigma-Aldrich), or 1 µM Jasplakinolide (Tocris) for 30 minutes at 37 °C. Erythroblasts were incubated with nocodazole (10 µM; Sigma) or taxol (paclitaxel, 10 µM; Sigma) at 37 °C for 3 hours. As indicated in the figure legends, cells were incubated with 30 µM Myristyl trimethyl ammonium bromide (MiTMAB; Calbiochem), 80 µM Dynasore (Sigma), 30 µM Pitstop-2 (Abcam) or 15 mM Sucrose (Sigma), for 15–45 minutes at 37 °C. All reagents were solubilised in DMSO (Sigma-Aldrich).

### Flow Cytometry

Expanding and differentiating erythroblasts, and K562 cells, were incubated with actin or microtubule disrupting drugs, and/or inhibitors of endocytosis, against a DMSO control. Cells were then washed in PBSAG before being labelled with extracellular primary antibody at 4 °C for 30 minutes. To separate erythroblasts from reticulocytes, cells were incubated with Hoechst 33342 (Sigma) for 45 minutes at 37 °C and fixed in 1% PFA and 0.0075% glutaraldehyde prior to labelling with primary antibody. The cells were washed and incubated for a further 30 minutes with rat allophycocyanin (APC)-conjugated anti-mouse IgG1 (Biolegend) for detection. Emissions from 10,000 events were collected using a MacsQuant Analyzer 10 flow cytometer and data was processed using FlowJo version X.10.6.

## Electronic supplementary material

Supplementary Figures, Table and legends
