# Supplementary material for: CD47 surface stability is sensitive to actin disruption prior to inclusion within the band 3 macrocomplex
Source: Sci Rep. 2017 May 22;7:2246. doi: 10.1038/s41598-017-02356-1 (PMC5440412; doi:10.1038/s41598-017-02356-1)
Supplement: Supplementary file 1 — Supplementary Figures, Table and legends [file 41598_2017_2356_MOESM1_ESM.pdf]

Supplement

CD47 surface stability is sensitive to actin disruption prior to inclusion within the band 3 macrocomplex

Kathryn E. Mordue[1], Bethan R. Hawley[1,2,3], Timothy J. Satchwell[1,2,3] & Ashley M. Toye[1,2,3]

[1]School of Biochemistry, Medical Sciences Building, University Walk, Bristol, UK;

[2]Bristol Institute of Transfusion Sciences, NHSBT, Filton, UK

[3]NIHR Blood and Transplant Unit in Red Blood Cell products at the University of Bristol.

Correspondenc

Supplementary Figure S1

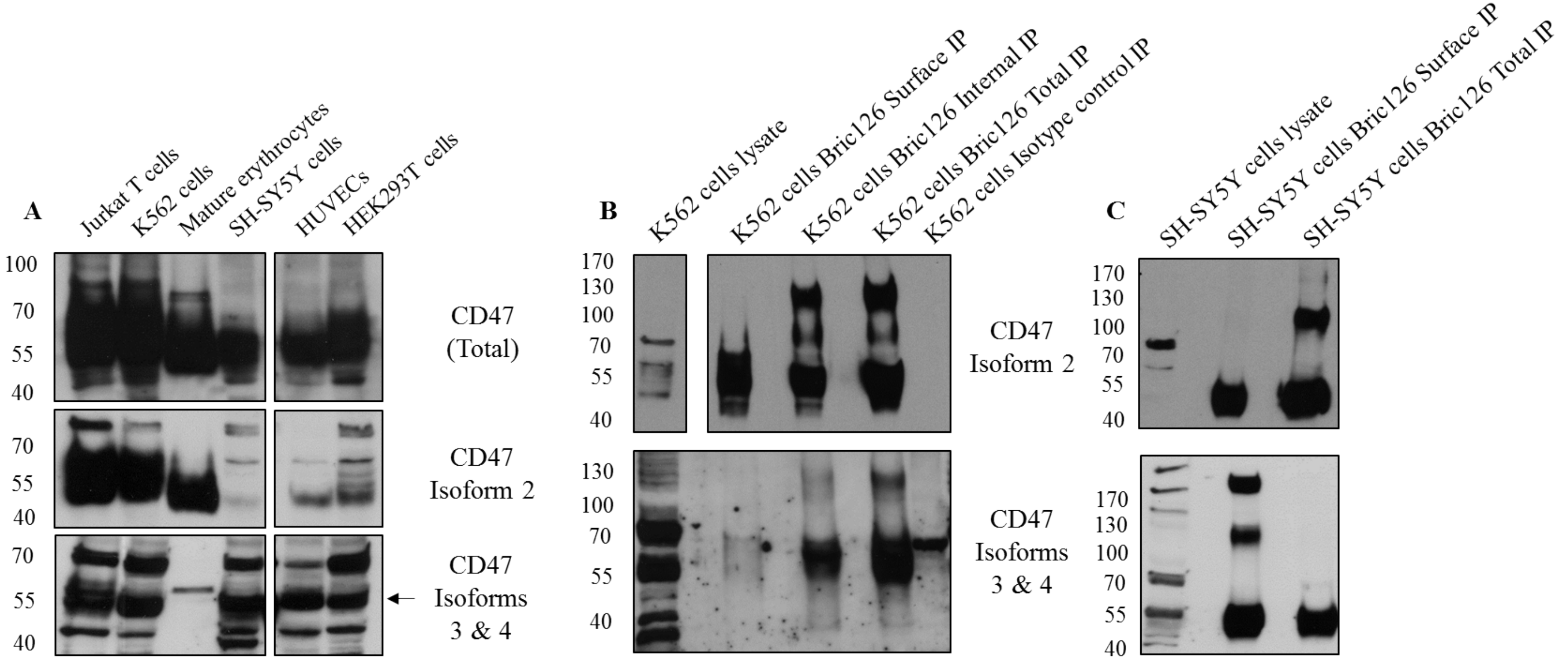

Figure S1. K562 cells and SH-SY5Y cells express CD47 isoform 2 and isoforms 3 and 4, but CD47 isoforms 3 and 4 were only detected at the surface of SH-SY5Y cells. (**A**) Cells were lysed and separated by SDS-PAGE (10^6 cells/lane). Western blots were then probed with anti-CD47out1 (Total), anti-CD47 isoform 2 or anti-CD47 isoforms 3 and 4 (protein band indicated by *) (n=2). Total cell, and cell surface immunoprecipitations followed by internal immunoprecipitations, were carried out on 5 - 10 x 10^6 K562 cells (**B**) and SH-SY5Y cells (**C**). The eluates were separated by SDS-PAGE against K562 and SH-SY5Y cell lysates (10^6 cells/lane). Western blots were then probed with anti-CD47 isoform 2 or anti-CD47 isoforms 3 and 4 (n=2).

Supplementary Figure S2.

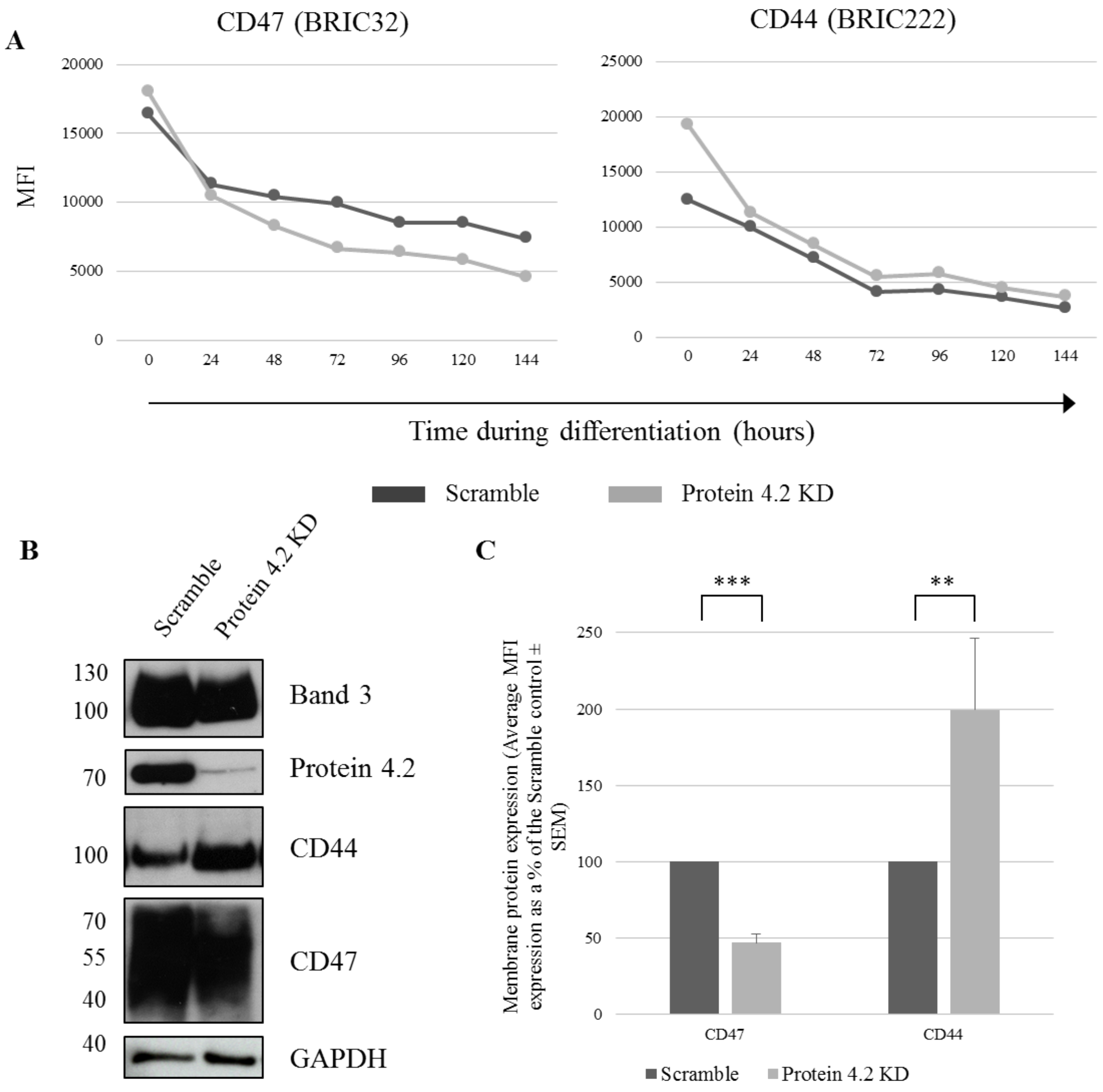

Figure S2 The hallmarks of protein 4.2 deficiency are recapitulated following shRNA-mediated knockdown of protein 4.2 via lentiviral transduction. Erythroblasts were transduced with lentiviral pLKO.1 protein 4.2 shRNA1, or a scramble shRNA control, as detailed in the methods. (**A** and **C**) 1.5 x 10^6 protein 4.2 deficient and scramble control erythroblasts were incubated with Hoechst for 45 minutes at 37°C, to separate erythroblasts from reticulocytes and nuclei, and fixed in 1% PFA and 0.0075% glutaraldehyde[24] prior to labelling with BRIC32 (CD47) or BRIC222 (CD44) for 30 minutes at 4°C. The cells were then incubated with an APC-conjugated monoclonal rat anti-mouse IgG1 secondary, and membrane protein expression was assessed by flow cytometry. (**A**) Every 24 hours during differentiation expression of CD47 and CD44 on erythroblasts was assessed by flow cytometry (n=3 for each time point). (**B**) Filtered reticulocytes generated from *in vitro* cultured protein 4.2 deficient erythroblasts compared to Scramble shRNA control erythroblast, were lysed and separated by SDS-PAGE (1.8 x 10^5 reticulocytes/lane). Western blots were probed with rabbit anti-C terminal band 3 antibody (YNTU), BRIC273 (anti-protein 4.2), BRIC222 (anti-CD44), anti-CD47 (CD47out1) and anti-GAPDH as a loading control (n=2). (**C**) Average protein expression of CD47 and CD44 on reticulocytes between 96-144 hours post-differentiation (Average MFI as a % of the scramble control ± SEM (n=3 at each time point), *** $p \leq 0.001$; ** $p \leq 0.01$ using the Students *T* test). Emissions from 10,000 events were detected using a MACSQuant Analyser 10 flow cytometer and data was analysed using FlowJo version 10.

Supplementary Figure S3

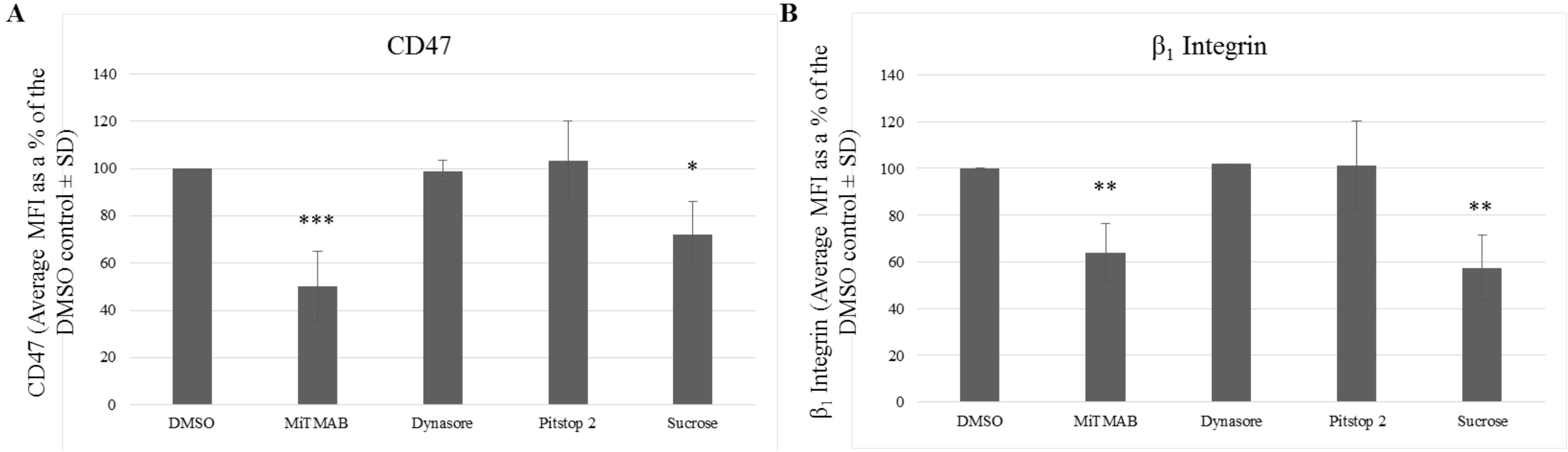

Figure S3. Inhibition of CD47 endocytosis in K562 cells. 1 x 10^6 K562 cells were incubated with MiTMAB (30μM), Dynasore (80μM), Pitstop 2 (30μM), Sucrose (15mM) or a DMSO control for 20 minutes at 37°C. K562 cells were then stained with (**A**) BRIC32 (anti-CD47), or (**B**) P5D2 (anti-β_1 Integrin) followed by APC-conjugated monoclonal IgG1 anti-mouse secondary antibody, before being assessed by flow cytometry (Average MFI as a % of the DMSO controls ± SD (n=4); *** $p \leq 0.001$; ** $p \leq 0.01$, * $p \leq 0.05$ using the Students *T* test). 10,000 events were detected using a MACSQuant Analyser 10 flow cytometer and data was analysed using FlowJo version 10.

Supplemental Table 1. Summary of the proteomic profile of peptides detected following BRIC126 immunoprecipitations (IP) in K562 cells, EXP, T0 and T48 erythroblasts, and mature erythrocytes (RBCs). The total BRIC126 IP peptides and IgG peptides are shown alongside the ratio total/IgG. Clean total (Tot) peptides are underlined and bold. * Indicates no ratio due to absence of IgG control peptides . Peptides detected in the BRIC126 IP that are less than 2-fold enriched, compared to the isotype control (IgG) are highlighted in grey.

Accession Number	Protein Description	K562 cells			EXP			T0			T48			Mature RBCs		
		Tot	IgG	Ratio	Tot	IgG	Ratio	Tot	IgG	Ratio	Tot	IgG	Ratio	Tot	IgG	Ratio
**P60709**	Actin, cytoplasmic 1	71	33	2.2	242	109	2.2	194	114	1.7	18	42	0.4	155	139	1.1
**P61158**	Actin-related protein 3	**5**	-	*	8	6	1.3	13	6	2.2	-	-	-	2	2	1
**O43707**	Alpha-actinin-4	**8**	-	*	59	20	3.0	86	45	2.0	-	-	-	25	29	0.9
**Q562R1**	β-actin like protein 2	14	3	4.7	45	19	2.4	42	25	1.7	-	-	-	35	39	0.9
**Q96C19**	EF hand containing protein D2	**7**	-	*	12	3	4	15	6	2.5	-	-	-	**6**	-	*
**P15311**	Ezrin	12	6	2	-	-	-	-	-	-	-	-	-	6	14	0.4
**P47756**	F-actin capping protein subunit β	7	1	7	17	6	2.8	12	9	1.3	-	-	-	3	3	1
**P21333**	Filamin A	42	15	2.8	54	6	9	17	25	0.7	-	-	-	20	62	0.3
**Q08722**	Leucocyte surface antigen, CD47	6	1	6	7	2	3.5	8	4	2	6	5	1.2	14	6	2.3
**Q9UHB6**	LIMA1	-	-	-	7	2	3.5	27	5	5.4	-	-	-	3	5	0.6
**P26038**	Moesin	22	7	3.1	3	1	3	3	5	0.6	2	7	0.3	4	23	0.2
**O94832**	Myosin-Id	12	2	6	20	12	1.7	34	19	1.8	-	-	-	20	37	0.5
**P35579**	Myosin-9	228	72	3.2	374	166	2.3	224	185	1.2	5	17	0.3	302	410	0.7
**P35580**	Myosin-10	154	37	4.2	240	132	1.8	204	172	1.2	2	7	0.3	196	284	0.7

**P12829**	Myosin light chain 4	13	2	6.5	8	1	8	26	5	5.2	1	1	1	19	25	0.8
**P35241**	Radixin	10	5	2	-	-	-	-	-	-	-	-	-	-	-	-
**P02549**	Spectrin α chain, erythrocyte	-	-	-	**19**	-	*	30	27	1.1	1	5	0.2	-	-	-
**P11277**	Spectrin β chain, erythrocyte	-	-	-	9	1	9	27	23	1.2	-	-	-	-	-	-
**Q9NYL9**	Tropomodulin-3	-	-	-	**8**	-	*	8	2	4	-	-	-	2	1	2
**P06753**	Tropomyosin α-3 chain	9	1	9	3	3	1	**5**	-	*	-	-	-	8	5	1.6
**Q71U36**	Tubulin α-1A chain	25	17	1.5	**14**	-	*	**23**	-	*	11	28	0.4	-	-	-
**P62988**	Ubiquitin	19	3	6.3	2	2	1	4	3	1.3	7	9	0.8	4	20	0.2
**P68036**	Ubiquitin-conjugated enzyme E2 L3	14	5	2.8	-	-	-	-	-	-	-	-	-	**4**	-	*
**P08670**	Vimentin	-	-	-	**5**	-	*	**2**	-	*	-	-	-	10	13	0.8
